# Supplementary material for: Evaluating the ability of an artificial-intelligence cloud-based platform designed to provide information prior to locoregional therapy for breast cancer in improving patient’s satisfaction with therapy: The CINDERELLA trial
Source: PLoS One. 2023 Aug 3;18(8):e0289365. doi: 10.1371/journal.pone.0289365 (PMC10399739; doi:10.1371/journal.pone.0289365)
Supplement: S1 Checklist — (DOCX) [file pone.0289365.s001.docx]

**Table 1 | SPIRIT-AI checklist**

| **Section** | **Item** | **SPIRIT 2013 item** | **SPIRIT-AI item** | **Page**  **Number** |
| --- | --- | --- | --- | --- |
| Administrative Information | | | | |
| **Title** | 1 | Descriptive title identifying the study design, population, interventions, and, if applicable, trial acronym | SPIRIT-AI elaboration  Indicate that the intervention involve AI /ML specify the type model | 1 |
|  |  |  | SPIRIT-AI elaboration  Specify the intended use of the AI intervention | TITLE page |
| **Trial registration** | 2a | Trial identifier and registry name | Clinical Trials.gov Identifier 05196269 | 2 |
|  | 2b | All items from WHO Trial Registration Dataset |  | 2 |
| **Protocol version** | 3 | Date and version identifier |  | 2 |
| **Funding** | 4 | Sources and type | HORIZON-RIA. Proposal number 101057389 | 2 |
| **Roles and responsabilities** | 5a | Names, affiliations, and roles of protocol contributors |  | 1 |
|  | 5b | Name and contact information for the trial sponsor | Not applicable |  |
|  | 5c | Role of sand funders, if any, in study design; collection, management, analysis, and  interpretation of data; writing of the report; and the decision to submit the report for publication,  including whether they will have ultimate authority over any of these activities | HORIZON-RIA. Proposal number 101057389 | EU funding reference |
|  | 5d | Composition, roles, and responsibilities of the coordinating center, steering committee, endpoint adjudication committee, data management team, and other individuals or groups overseeing  the trial, if applicable (see Item 21a for data monitoring committee) | HORIZON-RIA. Proposal number 101057389 | Grant and Consortium Agreement |
| Introduction | | | | |
| **Background and rationale** | 6a | Description of research question and justification for undertaking the trial, including summary of  relevant studies (published and unpublished) examining benefits and harms for each intervention | SPIRIT-AI Extension  Explain the intended use of the AI  intervention in the context of the  clinical pathway, including its purpose  and its intended users (for example,  healthcare professionals, patients,  public). | 5 |
|  | 6b | Explanation for choice of comparators | SPIRIT-AI Extension  Describe any pre-existing evidence  for the AI intervention.  (No previous comparators) | NA |
| **Objectives** | 7 | Specific objectives or hypothesis | Table 1 |  |
| **Trial design** | 8 | Description of trial design including type of trial (for example, parallel group, crossover, factorial,  single group), allocation ratio, and framework (for example, superiority, equivalence, noninferiority, exploratory) | Done | 7 |
| Methods: participants, interventions and outcomes | | | |  |
| **Study setting** | 9 | Description of study settings (for example, community clinic, academic hospital) and list of countries where data will be collected. Reference to where list of study sites can be obtained | SPIRIT-AI Extension  Describe the onsite and offsite  requirements needed to integrate the  AI intervention into the trial setting. | 7 |
| **Eligibility criteria** | 10 | Inclusion and exclusion criteria for participants. If applicable, eligibility criteria for study centers and individuals who will perform the interventions (for example, surgeons, psychotherapists) | SPIRIT-AI Extension  State the inclusion and exclusion  criteria at the level of participants. | 7,8 |
|  |  |  | SPIRIT-AI Extension  State the inclusion and exclusion  criteria at the level of the input data. | 7,8 |
| **Interventions** | 11a | Interventions for each group with sufficient detail to allow replication, including how and when they will be administered | SPIRIT-AI Extension  State which version of the AI  algorithm will be used. | WEB BCCTCore V1.0 |
|  |  |  | SPIRIT-AI Extension  Specify the procedure for acquiring  and selecting the input data for the AI  intervention. | Digital Photographs of Patients  Torso |
|  |  |  | SPIRIT-AI Extension  Specify the procedure for assessing  and handling poor-quality or unavailable input data. | All images evaluation will be centralized on the same platform and ideally captured with a photorobot |
|  |  |  | SPIRIT-AI Extension  Specify whether there is human–AI  interaction in the handling of the input data, and what level of expertise is required for users. | 11 |
|  |  |  | SPIRIT-AI Extension  Specify the output of the AI intervention. | 13 |
|  |  |  | SPIRIT-AI Extension  Explain the procedure for how the AI  intervention’s output will contribute  to decision-making or other elements  of clinical practice. | 12 |
|  | 11b | Criteria for discontinuing or modifying allocated interventions for a given trial participant (for  example, drug dose change in response to harms, participant request, or improving/worsening  disease) | NA | NA |
|  | 11c | Strategies to improve adherence to intervention protocols, and any procedures for monitoring  adherence (for example, drug tablet return, laboratory tests) | Not applicable | NA |
|  | 11d | Relevant concomitant care and interventions that are permitted or prohibited during the trial | Not applicable | NA |
| **Outcomes** | 12 | Primary, secondary, and other outcomes,  including the specific measurement variable analysis metric, method of aggregation, and time point for each outcome. Explanation of the clinical relevance of chosen efficacy and harm outcomes is strongly recommended | Table 4 | Table 4 |
| **Participant timeline** | 13 | Time schedule of enrollment, interventions  and visits for participants. A schematic  diagram is highly recommended. | Table 2 | Table 2 |
| **Sample size** | 14 | Estimated number of participants needed  to achieve study objectives and how it was  determined, including clinical and statistical  assumptions supporting any sample size  calculations |  | 7 |
| **Recruitment** | 15 | Strategies for achieving adequate participant enrollment to reach target sample size |  | 11 |

| Methods: assignement of interventions (for controlled trials) | | | | |
| --- | --- | --- | --- | --- |
| **Sequence generation** | 16a | Method of generating the allocation sequence (for example, computer-generated random numbers), and list of any factors for stratification.  To reduce predictability of a random sequence, details of any planned restriction (for example, blocking) should be provided in a separate document that is unavailable to those who enroll participants or assign interventions |  | 8 |
| **Allocation , concealment mechanism** | 16b | Mechanism of implementing the allocation  sequence (for example, central telephone;  sequentially numbered, opaque, sealed  envelopes), describing any steps to conceal the sequence until interventions are assigned |  | 8 |
| **Implementation** | 16c | Who will generate the allocation sequence,  who will enroll participants, and who will assign participants to interventions |  | 8 |
| **Blinding** | 17a | Who will be blinded after assignment to interventions (for example, trial participants, care providers, outcome assessors, data analysts), and how |  | NA |
|  | 17b | If blinded, circumstances under which unblinding is permissible, and procedure for revealing a participant’s allocated intervention during the trial |  | NA |
| Methods: data collection, management and analysis | | | | |
| **Data collection methods** | 18a | Plans for assessment and collection of outcome, baseline, and other trial data, including any related processes to promote data quality (for example, duplicate measurements, training of assessors) and a description of study instruments  (for example, questionnaires, laboratory tests) along with their reliability and validity, if known.  Reference to where data collection forms can be found, if not in the protocol |  | 8 |
|  | 18b | Plans to promote participant retention and  complete follow-up, including list of any outcome data to be collected for participants who discontinue or deviate from intervention protocols |  | 8 |
| **Data management** | 19 | Plans for data entry, coding, security, and storage, including any related processes to promote data quality (for example, double data entry; range checks for data values). Reference to where details of data management procedures can be  found, if not in the protocol |  | 10 |
| **Statistical methods** | 20a | Statistical methods for analyzing primary and secondary outcomes. Reference to where other details of the statistical analysis plan can be found, if not in the protocol |  | 8,9 |
|  | 20b | Methods for any additional analyses (for example, subgroup and adjusted analyses) |  | 9 |
|  | 20c | Definition of analysis population relating to protocol non-adherence (for example, as randomized analysis), and any statistical methods to handle missing data (for example, multiple imputation) |  | 9 |
| Methods: monitoring | | | | |
| **Data monitoring** | 21a | Composition of data monitoring committee  (DMC); summary of its role and reporting  structure; statement of whether it is independent from the sponsor and competing interests; and reference to where further details about its charter can be found, if not in the protocol.  Alternatively, an explanation of why a DMC is not needed |  | EU |
|  | 21b | Description of any interim analyses and stopping guidelines, including who will have access to these interim results and make the final decision to terminate the trial |  | Grant and Consortium Agreement |
| **Harms** | 22 | Plans for collecting, assessing, reporting, and managing solicited and spontaneously reported adverse events and other unintended effects of trial interventions or trial conduct | SPIRIT-AI Extension  Specify any plans to identify and  analyze performance errors. If there  are no plans for this, justify why not. | Currently in development  Phase. Part of the EU requirements |
| **Auditing** | 23 | Frequency and procedures for auditing trial  conduct, if any, and whether the process will be independent from investigators and the sponsor |  | EU determined |
| Ethics and dissemination | | | | |
| **Research ethics approval** | 24 | Plans for seeking research ethics committee/ institutional review board (REC/IRB) approval |  | 7 |
| **Protocol amendments** | 25 | Plans for communicating important protocol modifications (for example, changes to eligibility criteria, outcomes, analyses) to relevant parties (for example, investigators, REC/IRBs, trial participants, trial registries, journals, regulators) |  | 7 |
| **Consent or ascent** | 26a | Who will obtain informed consent or assent  from potential trial participants or authorized surrogates, and how (see Item 32) |  | NA |
|  | 26b | Additional consent provisions for collection and use of participant data and biological specimens in ancillary studies, if applicable |  | NA |
| **Confidentiality** | 27 | How personal information about potential and enrolled participants will be collected, shared, and maintained in order to protect confidentiality before, during, and after the trial |  | 10 |
| **Declaration of interests** | 28 | Financial and other competing interests for  principal investigators for the overall trial and each study site |  | NA |
| **Access to data** | 29 | Statement of who will have access to the final trial dataset, and disclosure of contractual agreements that limit such access for investigators | SPIRIT-AI Extension  State whether and how the AI intervention and/or its code can be accessed, including any restrictions to access or re-use. | Grant and Consortium Agreement |
| **Ancillary and post-trial care** | 30 | Provisions, if any, for ancillary and post-trial care, and for compensation to those who suffer harm from trial participation |  | NA |
| **Dissemination policy** | 31a | Plans for investigators and sponsor to  communicate trial results to participants,  healthcare professionals, the public, and other relevant groups (for example, via publication, reporting in results databases, or other data sharing arrangements), including any publication restrictions |  | YES |
|  | 31b | Authorship eligibility guidelines and any intended use of professional writers |  | Grant and Consortium Agreement |
|  | 31c | Plans, if any, for granting public access to the full protocol, participant-level dataset, and statistical code |  | Grant and Consortium Agreement |
| Appendices | | | | |
| **Informed consent materials** | 32 | Model consent form and other related  documentation given to participants and  authorized surrogates |  | YES |
| **Biological specimens** | 33 | Plans for collection, laboratory evaluation, and storage of biological specimens for genetic or molecular analysis in the current trial and form future use in ancillary studies, if applicable |  | NA |
