# Supplementary material for: Evaluating the ability of an artificial-intelligence cloud-based platform designed to provide information prior to locoregional therapy for breast cancer in improving patient’s satisfaction with therapy: The CINDERELLA trial
Source: PLoS One. 2023 Aug 3;18(8):e0289365. doi: 10.1371/journal.pone.0289365 (PMC10399739; doi:10.1371/journal.pone.0289365)
Supplement: S1 File — (DOCX) [file pone.0289365.s002.docx]

Ethics Committee of the Medical Faculty HD ÿ Alte Glockengiesserei 11/1 ÿ 69115 Heidelberg

Prof. Dr. medical Jörg Heil University Hospital Heidelberg Women's Hospital, Breast Center Im Neuenheimer

Feld 440 69120 Heidelberg

# Heidelberg University Ethics Committee of the Medical Faculty

Old bell foundry 11/1 69115 Heidelberg

Tel. +49 6221 56264-60 (head office)

Fax +49 6221 56264-80

[ethikkommission-I@med.uni-heidelberg.de](mailto:ethikkommission-I@med.uni-heidelberg.de)

04/20/2023

ts-sn/cd

# PROFESSIONAL ADVICE

[www.medical-faculty-hd.uni](http://www.medical-faculty-hd.uni/)

heidelberg.de/einrichtungen/zentrale einrichtungen/ethics committee

**Chair:**

***Our reference:* S-118/2023 (Please always state)**

Prof. Dr. medical dr hc Thomas Strowitzki

***Title:***

# Clinical validation of an AI-based approach to improve the shared decision-making process and outcomes in

**Breast Cancer Patients proposed for Locoregional treatment (CINDERELLA)**

**deputy Chair:**

Prof. Dr. medical Klaus Herfarth

**Management:** Dr.

medical Verena Pfeilschifter

Dear Professor Heil,

the ethics committee discussed your research project at the meeting on March 13, 2023. The additional requested information was received on 03/31/2023.

# The ethics committee has no objections to the conduct of the study.

However, it makes the following recommendations and notes:

# General: 1.

It is pointed out that future questions/studies based on the samples/data used in this study must be submitted to the ethics committee as new applications if the current application does not include these aspects.

2. In its assessment, the ethics committee assumes that only routine questionnaires will be used in the study. If study-specific questionnaires are used, these must be submitted later.

Information leaflet for patients: 3rd

p. 3 section "How is the study going?": The participants should be informed about the probability of being assigned to one of the two groups.

4. p. 5, section "Privacy Policy" a.

see "including genetic data (if your tumor has been genetically analyzed)...": The participants will only be informed at this point that their genetic data will also be processed for study purposes. However, it is not clear why this data is needed in the context of the study. The description of the course of the study on p. 3 does not suggest that, in addition to the results of the surveys and the

BIC SOLADEST600

IBAN DE64 6005 0101 7421 5004 29

.

Photographs further data, in particular genetic data, are to be processed. In the section "How is the study going?" the participants must also be informed about this (à Which data is specifically collected from the medical file for the study? Why is this data needed?).

b. According to the information in the "What happens to my data?" section, the data processed for the study should also be used to further train the artificial intelligence of the Cinderella app. To do this, the data, including the genetic data, would have to be passed on to the manufacturer of the app. The participants must be explicitly informed of this (also in the section “Transfer/Recipient” on p. 6). In addition, for the purpose of passing on and analyzing genetic data, the references in the sample

text of the working group of medical ethics committees on information and consent to the use of biomaterials and associated data in biobanks should be taken into account as a formulation aid

(www.akek.de).

c. The term “pseudonymisation” should be explained as follows: “Pseudonymisation means that the data collected is marked with a number/letter code instead of name/date of birth etc. The data can then only be assigned to a specific person by specific authorized persons with additional information (so- called "key")." d. It must be stated when the personal data will be anonymised. It should be noted that according to Art. 89 (1) GDPR,

personal data must be anonymized as soon as this is possible for the research purpose ÿ principle of data minimization.

5. P. 6, section "Distribution/recipient, see "The sponsor [...] financial framework.": The explanation of the tasks of the "sponsor" should be corrected to the effect that the sponsor acts as the funder of the study and coordinates the scientific evaluation . Responsibility for the legal and ethical aspects of the conduct of studies according to § 15 BOÄ lies with the respective study director.
6. p. 8, section "Duration of storage of the data", see "The sponsor stores the data still available in this form indefinitely after the study has been completed or discontinued for the following reasons...". inaccurate. Specific information on the retention period must be given; if this is not possible, criteria for determining the retention period must be specified (cf. Art. 13 Para. 2 lit. a GDPR).

We wish you every success in conducting the study.

Please forward the result of the professional legal advice and the correspondence relevant to the study to all participating doctors in our area of responsibility.

Best regards

Prof. Dr. medical dr hc Thomas Strowitzki Chairman

**Investments**

Attachment

# General information: •

Changes in the organization and course of the study must be reported to the commission immediately, together with an assessment of the risk-benefit ratio. Both the **application number** and the **changed passages** should be **clearly marked** in the relevant documents , otherwise speedy processing is not possible. • A **benefit/risk assessment** with regard to the current

**Covid-19 pandemic** must be carried out **continuously and independently .** The benefit of conducting the study during

against the risk of new infections (of the study participants and the study staff as well as indirectly other people) during the current pandemic. The risk of infection, e.g. through study-related contacts between those involved or study-related travel expenses, must always be assessed in accordance with the current pandemic situation. If the study requires regular measures (regular visits, regular treatment/administration of drugs or similar), it must also be considered whether and, if so, to what extent these measures can be reliably carried out during the pandemic. Possible precautions to minimize risk must also be taken. If, based on the updated benefit/risk assessment, measures have to be taken due to the pandemic and this leads to changes in the conduct of the study, these changes (in particular to the test plan and information sheet or declaration of consent) must be reported to the ethics committee in accordance with Section 10

(1) GCP-V as Subsequent change to be submitted. It is expressly pointed out that all study participants must be informed in writing and without delay about all changes in the study process that are relevant to them. § 11 of the GCP-V regulates the measures that may have to be taken immediately to protect against immediate danger. It is advisable to document all deviations from the protocol that can be attributed to the pandemic situation. In addition, the information of the Working Group on Medical Ethics Committees in the Federal Republic of Germany eV at [www.akek.de](http://www.akek.de/) must always be observed.
- Any research project involving test subjects is prior to the recruitment of the to register the first test subject in a publicly accessible database.
- Within one year of the end of the study, the study management should submit to the Commission a final report summarizing the results and conclusions of the study, whether fully completed or terminated early.

For this purpose, the “Final report” template available on the Commission's website should be used (path: Studies **ÿ** Other studies **ÿ** Application documents & templates).

- Aspects of research projects related to data protection law are generally only examined cursorily by the ethics committee. This vote / this assessment does not replace the consultation of the responsible data protection officer. Compliance with the relevant data protection laws and the implementation of the data protection concept are the responsibility of the study director/investigator or sponsor.
- The ethics committee assumes that, in the case of video visits, the requirements of Annex 31b of the Federal Framework Agreement are observed and the visits are carried out by a certified provider. • With the judgment of the

European Court of Justice of July 16, 2020 [file number C-311/18], the regulations of the EU-US Privacy Shield, in particular against the background of the Clarifying Lawful Overseas Use of Data Act (CLOUD Act) and the Foreign Surveillance Act (FISA) no longer constitutes a suitable legal framework. The persons responsible should therefore check in each individual case to what extent personal/personally identifiable data (i.e. also in the sense of Art. 4 Para. 5 DSGVO pseudonymised data sets) are legally secure either on the basis of suitable guarantees (such as Company rules, standard contractual clauses or on the basis of express consent after risk clarification pursuant to Article 49 (1) (a) GDPR).

It is advisable to carefully monitor the effects of the judgment and the likely subsequent regulatory guidelines on the part of the responsible authorities, particularly with regard to the standard contractual clauses.

- The ethics committee of the Heidelberg Medical Faculty works in accordance with national legal provisions and the ICH-GCP guidelines. Their deliberations are based on the declaration of the World Medical Association of Helsinki in the current version.
- Irrespective of the result of the consultation, the ethics committee draws your attention to the fact that the ethical and legal responsibility for conducting a study lies with the leader of the study and all participating physicians.

**Attachment**

**List of submitted documents**

**Primary submitted Documents:**

**content**

**Subsequent submission:**

Cover letter dated February 21, 2023 Summary

checklist for other studies Initial

application form

Information letter for patients Version 1 dated February 21, 2023 Declaration of consent Version 1 dated February 21,

2023 Clinical Study Protocol Version 1.3 dated August

5th, 2022 Prof. Jörg Moritz Heil’s CV dated

February 24th, 2023 Opinion on the Conselho ethics committee de Ética da Fundação Champalimaud Comissão de Ética para a Saude, Portugal from

May 24th, 2022 Use of CANKADO in Cinderella, Kraft GmbH AG Munich (without version number, undated)

CRF Version 1.0 of March 2022

Subsequent submission of March

31, 2023: Information sheet for patients Version 2 of March 31, 2023 (with and without marking the changes)

Declaration of consent version 2 from March 31, 2023 (with and without marking the changes)

Addendum to the CINDERELLA Protocol Version 1.3 from August 5th, 2022

Cinderella – Expectation Questionnaire Version 1.1 from March 31st, 2023 Consortium Agreement Version 2.0
